# Identifying Effective Components of a Social Marketing Campaign to Improve Engagement With Express Sexual Health Services Among Gay, Bisexual, and Other Men Who Have Sex With Men: Case Study

**DOI:** 10.2196/50944

**Published:** 2024-08-23

**Authors:** Laura C Chambers, Yelena Malyuta, William C Goedel, Philip A Chan, Cassandra Sutten Coats, Ken Allen, Amy S Nunn

**Affiliations:** 1 Division of Infectious Diseases, The Miriam Hospital, Providence, RI, United States; 2 Department of Epidemiology, School of Public Health, Brown University, Providence, RI, United States; 3 Open Door Health, Rhode Island Public Health Institute, Providence, RI, United States; 4 Department of Behavioral and Social Sciences, School of Public Health, Brown University, Providence, RI, United States; 5 Department of Medicine, Warren Alpert Medical School, Brown University, Providence, RI, United States; 6 The Allen Company, Inc, Jackson, MS, United States

**Keywords:** social marketing, sexually transmitted infection, HIV, sexual and gender minorities, sexual health, gay, MSM, men who have sex with men, STI, testing, digital marketing, digital, campaign, promote, treatment, prevention, bisexual, advertisement, Google display, Grindr, Facebook

## Abstract

**Background:**

Little is known about how best to reach people with social marketing messages promoting use of clinical HIV and sexually transmitted infection (STI) services.

**Objective:**

We evaluated a multiplatform, digital social marketing campaign intended to increase use of HIV/STI testing, treatment, and prevention services among gay, bisexual, and other men who have sex with men (MSM) at an LGBTQ+ (lesbian, gay, bisexual, transgender, queer, and/or questioning) community health center.

**Methods:**

We evaluated engagement with a social marketing campaign launched by Open Door Health, the only LGBTQ+ community health center in Rhode Island, during the first 8 months of implementation (April to November 2021). Three types of advertisements encouraging use of HIV/STI services were developed and implemented on Google Search, Google Display, Grindr, and Facebook. Platforms tracked the number of times that an advertisement was displayed to a user (impressions), that a user clicked through to a landing page that facilitated scheduling (clicks), and that a user requested a call to schedule an appointment from the landing page (conversions). We calculated the click-through rate (clicks per impression), conversion rate (conversions per click), and the dollar amount spent per 1000 impressions and per click and conversion.

**Results:**

Overall, Google Search yielded the highest click-through rate (7.1%) and conversion rate (7.0%) compared to Google Display, Grindr, and Facebook (click-through rates=0.4%‐3.3%; conversion rates=0%‐0.03%). Although the spend per 1000 impressions and per click was higher for Google Search compared to other platforms, the spend per conversion—which measures the number of people intending to attend the clinic for services—was substantially lower for Google Search (US $48.19 vs US $3120.42-US $3436.03).

**Conclusions:**

Campaigns using the Google Search platform may yield the greatest return on investment for engaging MSM in HIV/STI services at community health clinics. Future studies are needed to measure clinical outcomes among those who present to the clinic for services after viewing campaign advertisements and to compare the return on investment with use of social marketing campaigns relative to other approaches.

## Introduction

As of 2020, there were nearly 1.1 million people known to be living with HIV infection in the United States [1], and the rate of sexually transmitted infection (STI) diagnoses increased by 7% from 2017 to 2021 [2]. Although gay, bisexual, and other men who have sex with men (MSM) are estimated to represent only 4%‐6% of men in the United States [3], they represented 72% of men newly diagnosed with HIV in 2020 [1] and 36% of gonorrhea cases as well as primary and secondary syphilis cases in 2021 [2]. Additionally, while the annual number of new HIV infections diagnosed among MSM in the United States has decreased since 2017, substantial racial/ethnicity disparities in HIV diagnosis [4] and HIV pre-exposure prophylaxis (PrEP) access [5] persist. As such, efforts to achieve the national goal of reducing HIV incidence by at least 90% by 2030 [6] will be unsuccessful without reducing the incidence among MSM, and especially MSM of color, through comprehensive engagement with biomedical HIV prevention and treatment services.

Several social media platforms have been widely used by health departments and community-based organizations to disseminate health information, engage with MSM, and promote uptake of HIV and STI prevention and treatment services [7-12]. However, little is known about what messages work—and through which platforms—for generating appropriate levels of user engagement, diffusion of information across social networks [13], and ultimately, uptake of clinical services. Consistent with these challenges, researchers have sought to identify predictors of user engagement with digital content [14-17]. Studies suggest that impactful social marketing campaigns will contain positive sentiments, content about PrEP and mental health, and understandable information regarding intervention effectiveness, while avoiding solicitation of direct engagement by asking questions, posting during or after typical business hours, and content about dating [15,18-20]. However, little implementation research has been conducted into understanding how people engage with and react to social marketing messages [13].

Given that many public health organizations have limited marketing budgets but serve populations with great need, it is important to understand which social media platforms are most suitable for engaging MSM digitally. Understanding which platforms yield the greatest return on investment is also important for promoting uptake of HIV/STI services among people at highest risk of infection. To help fill this knowledge gap, we evaluated a multiplatform, digital social marketing campaign implemented by an LGBTQ+ (lesbian, gay, bisexual, transgender, queer, and/or questioning) community health center to increase utilization of express HIV/STI services at the clinic.

## Methods

### Setting

Open Door Health implemented the social marketing campaign in Providence, the capital city of Rhode Island. Open Door Health is the first and only LGBTQ+ community health center in the state and is located in the Providence ZIP code with the highest incidence of new HIV diagnoses. In Rhode Island, most new HIV diagnoses are among MSM and residents of Providence County. Among MSM, the majority of new HIV diagnoses are among those in their 20s and 30s, with young Black/African American and Hispanic/Latino MSM increasingly affected [21].

### Timeline

Open Door Health launched the social marketing campaign in April 2021. We evaluated engagement with the campaign across platforms during the first 8 months of implementation (April to November 2021). For context, in 2021, residents aged 16 years and older statewide became eligible for COVID-19 vaccination in mid-April, many restrictions on businesses and gatherings were eased in early May, and most restrictions on businesses and vaccinated residents were lifted by late May.

### Population

The goal of the social marketing campaign was to increase utilization of express HIV and STI services at Open Door Health. The clinic specializes in providing primary health care for the state’s sexual and gender minority communities. Open Door Health also provides comprehensive sexual health care, including HIV and STI screening, prevention, and treatment services. Patients without symptoms or a known or suspected exposure to infection may access “express” HIV/STI services, which include HIV/STI screening without a physical examination and are provided on a walk-in basis or with a scheduled appointment.

### Social Marketing Campaign

**Figure 1.**
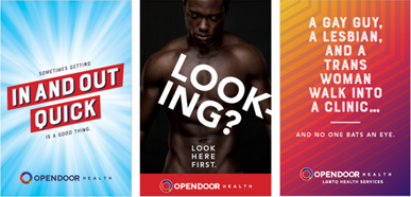
Open Door Health campaign advertisement types, including “Right Place,” “Got You Covered,” and “Punchline” (from left to right).

Campaign advertisements were developed by a marketing company, in consultation with clinic leadership and the community advisory board, based on the findings from a series of individual in-depth interviews and focus group discussions with local MSM (previously described in detail [22]). The campaign included three types of advertisements: “Right Place,” “Got You Covered,” and “Punchline” (Figure 1). Each advertisement type included multiple variations on the text, images, and format (Figures S1-S3 in Multimedia Appendix 1).

The advertisements were developed for and implemented on Google, Grindr, and Facebook. Instagram was not included because patients reported using Facebook at higher rates than Instagram. Google advertisements included two approaches: (1) search campaign advertisements that appeared as a text-based advertisement at the top of the results page for a keyword (Figure S4 in Multimedia Appendix 1) and (2) display campaign advertisements that appeared as combinations of headlines, images, and descriptive text on the Google Display Network, a collection of over 2 million partner websites where advertisements can appear as banners or sidebars while users browse other content. Google Display campaign advertisements were responsive (ie, automatically adjusted their size, appearance, and format to fit available space) and targeted to users based on a proprietary algorithm intended to maximize user engagement. Grindr and Facebook advertisements were a combination of headlines, images, and descriptive text targeted to users based on location, demographics, interests, and other profile information. Grindr advertisements were displayed as small or medium banners or full-screen interstitials. Facebook advertisements were displayed in a sidebar on the website or in the desktop and mobile feeds as sponsored content. All platforms took people who clicked on the advertisement to a landing page specific to that advertisement type (Figures S5-S7 in Multimedia Appendix 1). Although the text-based advertisements of the Google Search campaign were not specific to one of the advertisement types, people who clicked on the advertisements were taken to one of the three landing pages.

Some platforms allowed for re-engagement of users who had viewed an advertisement but did not engage with it by clicking on it. In a process called retargeting, a piece of code attached an anonymous, undetectable browser cookie to every user who viewed an advertisement. After those users left the platform on which they originally viewed the advertisement, the cookie could trigger a digital advertising platform to show another advertisement on a different platform. For example, a user may have been presented with an advertisement on Grindr that they did not click on, so a Google Display campaign advertisement may then appear on other websites.

All three advertisement types were implemented on Google Search and Google Display; the “Right Place” and “Got You Covered” advertisement types were implemented on Grindr; and the “Right Place” and “Punchline” advertisement types were implemented on Facebook. Users were able to click through an advertisement to a landing page where they could request a phone call to schedule an appointment at the clinic.

### Measures

Platforms generally tracked the number of users to whom an advertisement was shown (reach), the number of times that an advertisement was displayed to a user (impressions), the number of times that a user clicked through an advertisement to a landing page where they could request a phone call to schedule an appointment (clicks), and the number of phone calls requested from that landing page (conversions). To measure campaign engagement for specific advertisement and platform combinations, we calculated (1) the click-through rate (defined as the number of clicks divided by the number of impressions), (2) the conversion rate (defined as the number of conversions divided by the number of clicks), and (3) the dollar amount spent per 1000 impressions and per click and conversion.

### Data Analyses

We summarized the reach, impressions, clicks, conversions, spend, click-through rate, conversion rate, spend per 1000 impressions, spend per click, and spend per conversion for the social marketing campaign by platform overall, by advertisement type, and by specific advertisement.

### Ethical Considerations

The Brown University Institutional Review Board determined that this secondary analysis of deidentified Open Door Health data did not meet the federal definition of human subjects research in Title 45 Code of Federal Regulations Part 46.102(e)(1), and therefore, review was not necessary (application number 3079).

## Results

The 8-month social media campaign implemented by Open Door Health in Providence, Rhode Island, included 3 types of advertisements tailored and implemented across 3 social media platforms, with US $29,711.84 in total spend and yielding a total of 2,536,405 impressions, 35,022 clicks, and 225 conversions across advertisement types and platforms. Considering all advertisement types combined, Google Search yielded the highest click-through rate (7.1%) and conversion rate (7.0%) compared to Google Display, Grindr, and Facebook (click-through rates=0.4%‐3.3%; conversion rates=0%‐0.03%; Table 1). Although spend per 1000 impressions and per click was higher for Google Search than other platforms, the spend per conversion was substantially lower for Google Search (US $48.19 vs US $3120.42‐US $3436.03). Of note, Grindr had the second highest click-through rate (3.3%) after Google Search, while Google Display had the lowest spend per 1000 impressions (US $4.87) and Grindr had the lowest spend per click (US $0.36).

**Table 1.** Open Door Health campaign results by advertisement type and social media platform (April 1 to November 30, 2021).

Ad type^a^ and measure		Platform			
		Google Search^b^	Google Display	Grindr	Facebook
**All**					
	Reach, n	Not measured	Not measured	Not measured	302,000
	Impressions, n	44,728	706,063	531,218	1,254,396
	Clicks, n	3180	2930	17,360	11,552
	Conversions, n	221	1	0	3
	Spend (US $)	10,650.21	3436.03	6264.33	9361.27
	Click-through rate (%)	7.11	0.41	3.27	0.92
	Conversion rate (%)	6.95	0.03	0.00	0.03
	Spend/1000 impressions (US $)	238.11	4.87	11.79	7.46
	Spend/click (US $)	3.35	1.17	0.36	0.81
	Spend/conversion (US $)	48.19	3436.03	Undefined	3,120.42
**Right place**					
	Reach, n	Not measured	Not measured	Not measured	229,227
	Impressions, n	38,628	211,401	266,910	1,019,206
	Clicks, n	2678	1049	8461	8454
	Conversions, n	195	0	0	2
	Spend (US $)	9297.41	1183.88	3129.22	6923.02
	Click-through rate (%)	6.93	0.50	3.17	0.83
	Conversion rate (%)	7.28	0.00	0.00	0.02
	Spend/1000 impressions (US $)	240.69	5.60	11.72	6.79
	Spend/click (US $)	3.47	1.13	0.37	0.82
	Spend/conversion (US $)	47.68	Undefined	Undefined	3461.51
**Got you covered**					
	Reach, n	Not measured	Not measured	Not measured	—^c^
	Impressions, n	3252	231,393	264,308	—
	Clicks, n	260	1348	8899	—
	Conversions, n	16	1	0	—
	Spend (US $)	708.58	1773.03	3135.11	—
	Click-through rate (%)	8.00	0.58	3.37	—
	Conversion rate (%)	6.15	0.07	0.00	—
	Spend/1000 impressions (US $)	217.89	7.66	11.86	—
	Spend/click (US $)	2.73	1.32	0.35	—
	Spend/conversion (US $)	44.29	1773.03	Undefined	—
**Punchline**					
	Reach, n	Not measured	Not measured	—	72,773
	Impressions, n	2848	263,269	—	235,190
	Clicks, n	242	533	—	3098
	Conversions, n	10	0	—	1
	Spend (US $)	644.22	479.12	—	2438.25
	Click-through rate (%)	8.50	0.20	—	1.32
	Conversion rate (%)	4.13	0.00	—	0.03
	Spend/1000 impressions (US $)	226.20	1.82	—	10.37
	Spend/click (US $)	2.66	0.90	—	0.79
	Spend/conversion (US $)	64.42	Undefined	—	2438.25

a All ads for each campaign were combined.

b Google Search campaign included text-only ads that were not specific to an ad type. However, people who clicked on the text-only ad were taken to a landing page designed for one of the ad types. Results are presented by landing page type.

c Did not run.

The overall pattern by social media platform was generally consistent for each of the advertisement types. However, it is important to keep in mind that the initial advertisements viewed through Google Search were text-based; it was the landing page for people who clicked on the text-based advertisements that was tailored to a specific advertisement type. The results for specific advertisements within each advertisement type are available in Tables S1-S3 in Multimedia Appendix 1.

## Discussion

In the first 8 months of a multiplatform digital social marketing campaign to increase utilization of express HIV/STI services at an LGBTQ+ community health center, Google Search yielded higher click-through and conversion rates (both about 7%) than Google Display, Grindr, and Facebook (0%‐3%). The cost of advertising per impression and click was notably higher for Google Search compared to other platforms; however, the cost per conversion—which measures the number of patients intending to present to the clinic for services—was markedly lower for Google Search (about US $48) than other platforms (more than US $3000). Grindr had the second highest click-through rate (about 3%), but none of the clicks resulted in requests for a call with the clinic.

Importantly, the goal of the social media campaign was to increase utilization of express HIV/STI services at Open Door Health. Although we were able to measure the number of patients intending to present for services, additional research on subsequent presentation to the clinic and services ultimately received is critical for further evaluating the impact of the campaign. It will also be important to evaluate the sociodemographics of the patients who present to the clinic as a result of each advertisement to learn whether certain advertisements resonate more with specific populations at high risk of HIV and STI, such as MSM of color. While overall engagement with the advertisements was similar across the advertisement types included in the campaign, the specific populations engaging with each advertisement type may have differed. Additionally, some advertisement types were not implemented on Grindr and Facebook due to limited initial user engagement with other advertisements on those platforms; it is possible these additional advertisements would have performed differently on those platforms.

In this study, we demonstrated a straightforward approach for calculating the return on investment for advertising dollars when designing tailored campaigns in the context of limited resources. Although Google Search yielded the greatest return on investment of the four platforms, the cost per conversion was relatively high (nearly US $50). However, the target population for the campaign is at highest risk for HIV acquisition in Rhode Island, and spending of up to US $379,668 to prevent one HIV infection is considered cost saving per US Centers for Disease Control and Prevention guidance [23]. In future studies, it will be important to measure clinical outcomes among those reached by the campaign who present to the clinic for services and to compare this return on investment with that of other approaches to increase uptake of HIV and STI prevention and treatment services to inform decisions regarding where to invest limited budgets.

In conclusion, social marketing campaigns using the Google Search platform, and thereby displaying the ads based on HIV- or STI-related search terms, may have the greatest return on investment for prompting phone calls with the clinic to schedule appointments for HIV/STI services, which we expect is most likely to result in presentation to the clinic for services. Although Grindr generated many initial clicks, this did not translate into phone calls with the clinic. However, engagement on Grindr likely increased awareness of the clinic that contributed to future retargeting and engagement on other platforms, such as Google. Thus, spending on Grindr and other platforms may be useful for improving reach and building community trust, which may subsequently improve the conversion rate through Google Search; future research in this area is needed.

## Supplementary material

10.2196/50944 Multimedia Appendix 1 Additional advertisement images and results by specific advertisement.

## Abbreviations

LGBTQ+: lesbian, gay, bisexual, transgender, queer, and/or questioning
MSM: men who have sex with men
PrEP: pre-exposure prophylaxis
STI: sexually transmitted infection
