# Supplementary material for: Identifying Effective Components of a Social Marketing Campaign to Improve Engagement With Express Sexual Health Services Among Gay, Bisexual, and Other Men Who Have Sex With Men: Case Study
Source: JMIR Public Health Surveill. 2024 Aug 23;10:e50944. doi: 10.2196/50944 (PMC11363878; doi:10.2196/50944)
Supplement: Multimedia Appendix 1 [file publichealth-v10-e50944-s001.docx]

## Multimedia Appendix

### Table of Contents

1. Figure S1: Specific advertisements within “Right Place” advertisement type
2. Figure S2: Specific advertisements within “Got You Covered” advertisements type
3. Figure S3: Specific advertisements within “Punchline” advertisements type
4. Figure S4: Example Google search campaign text-based advertisements
5. Figure S5: “Right Place” landing page
6. Figure S6: “Got You Covered” landing page
7. Figure S6: “Punchline” landing page
8. Table S1: Performance of specific advertisements within “Right Place” advertisements type
9. Table S2: Performance of specific advertisements within “Got You Covered” advertisements type
10. Table S3: Performance of specific advertisements within “Punchline” advertisements type

Figure S1. Specific advertisements within “Right Place” advertisement type


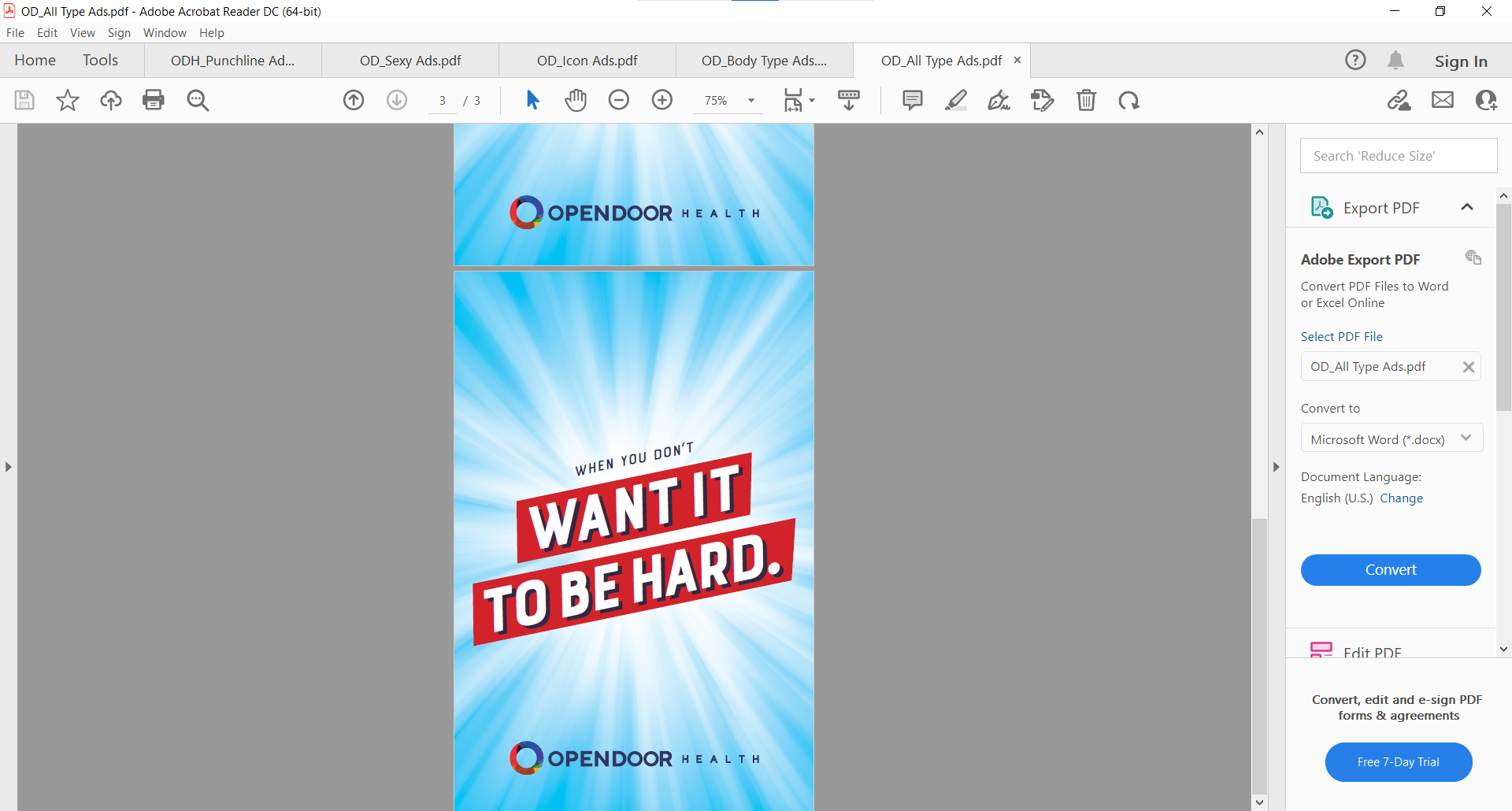

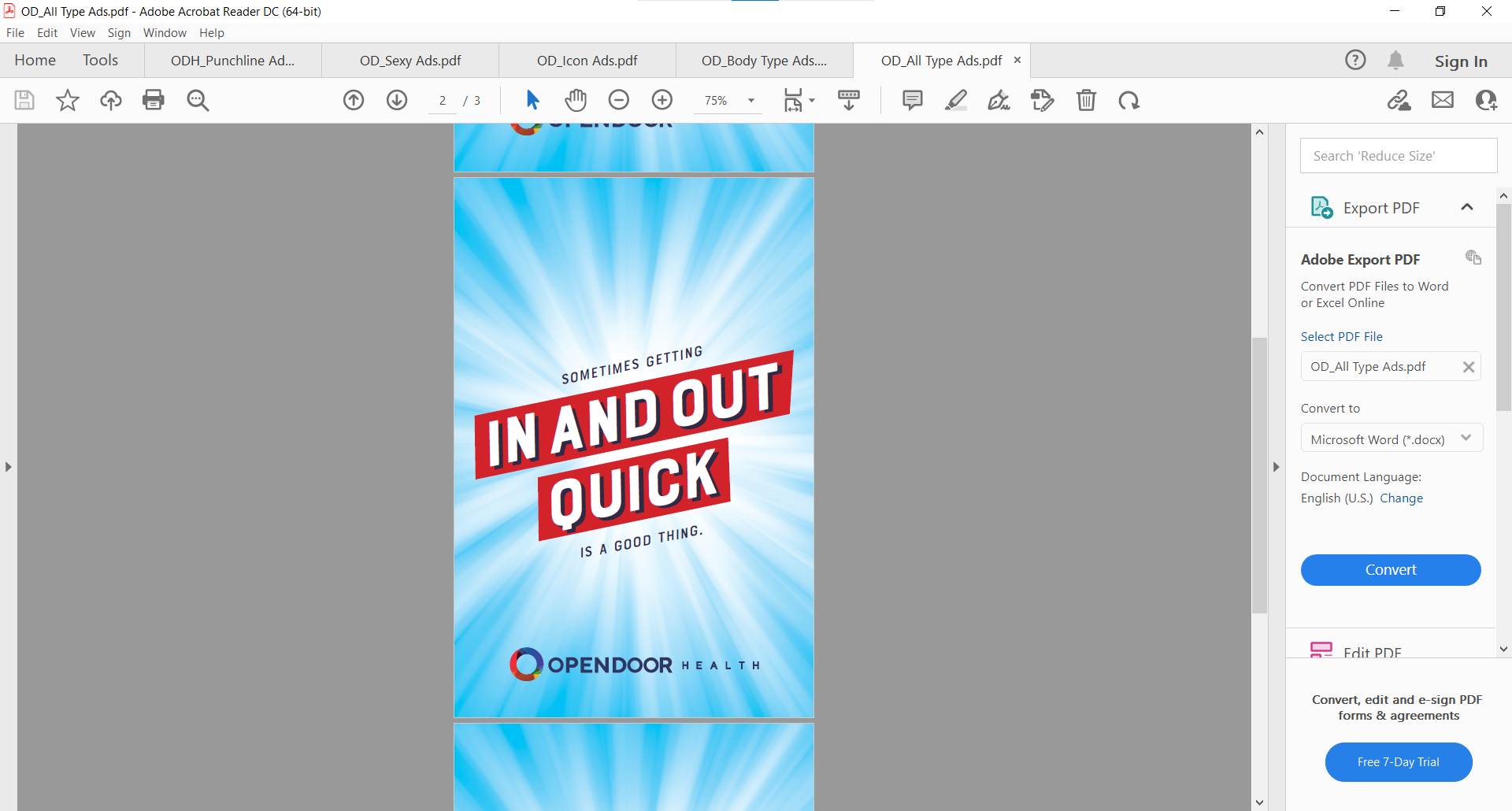


Figure S2. Specific advertisements within “Got You Covered” advertisement type


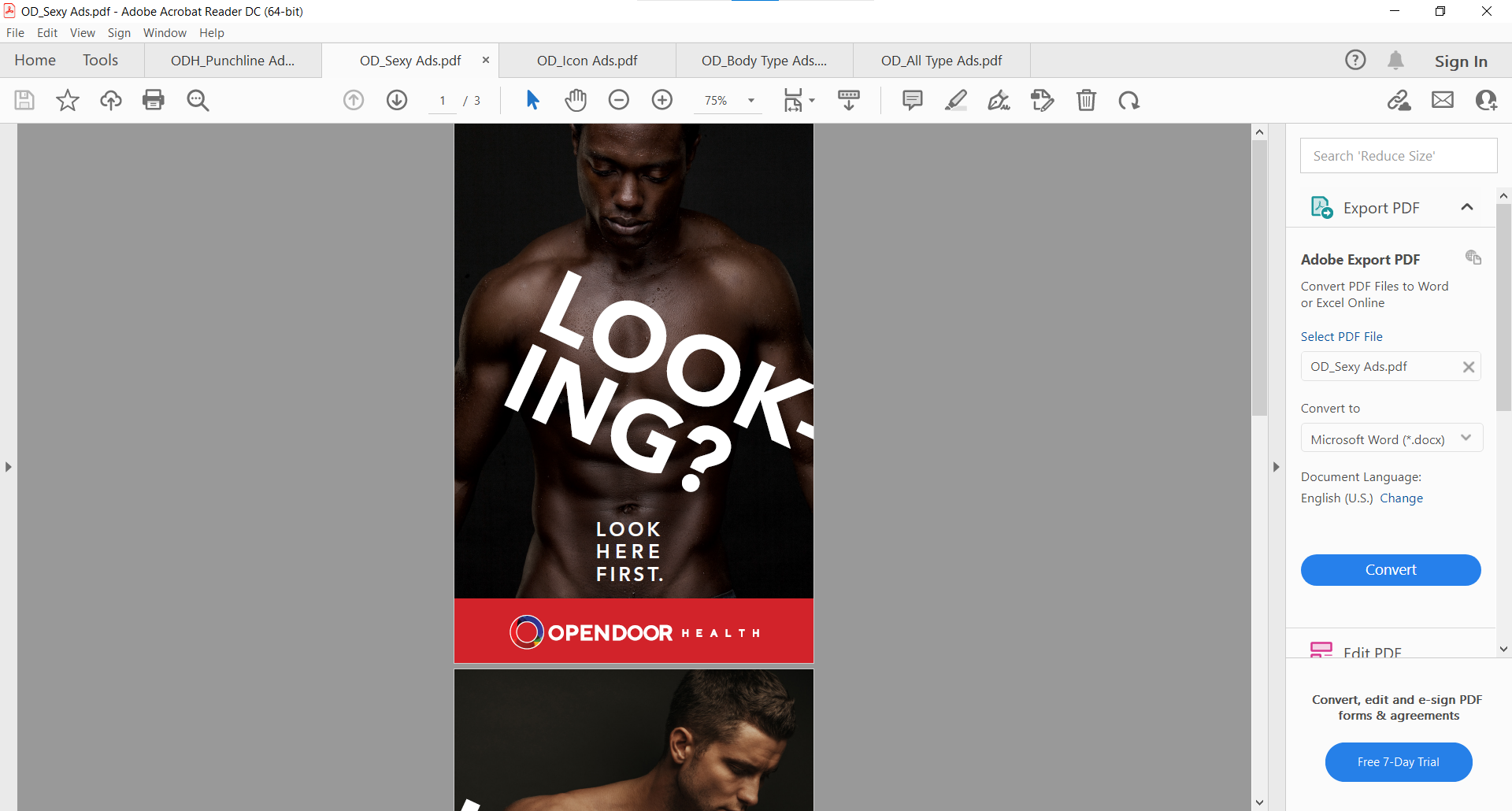

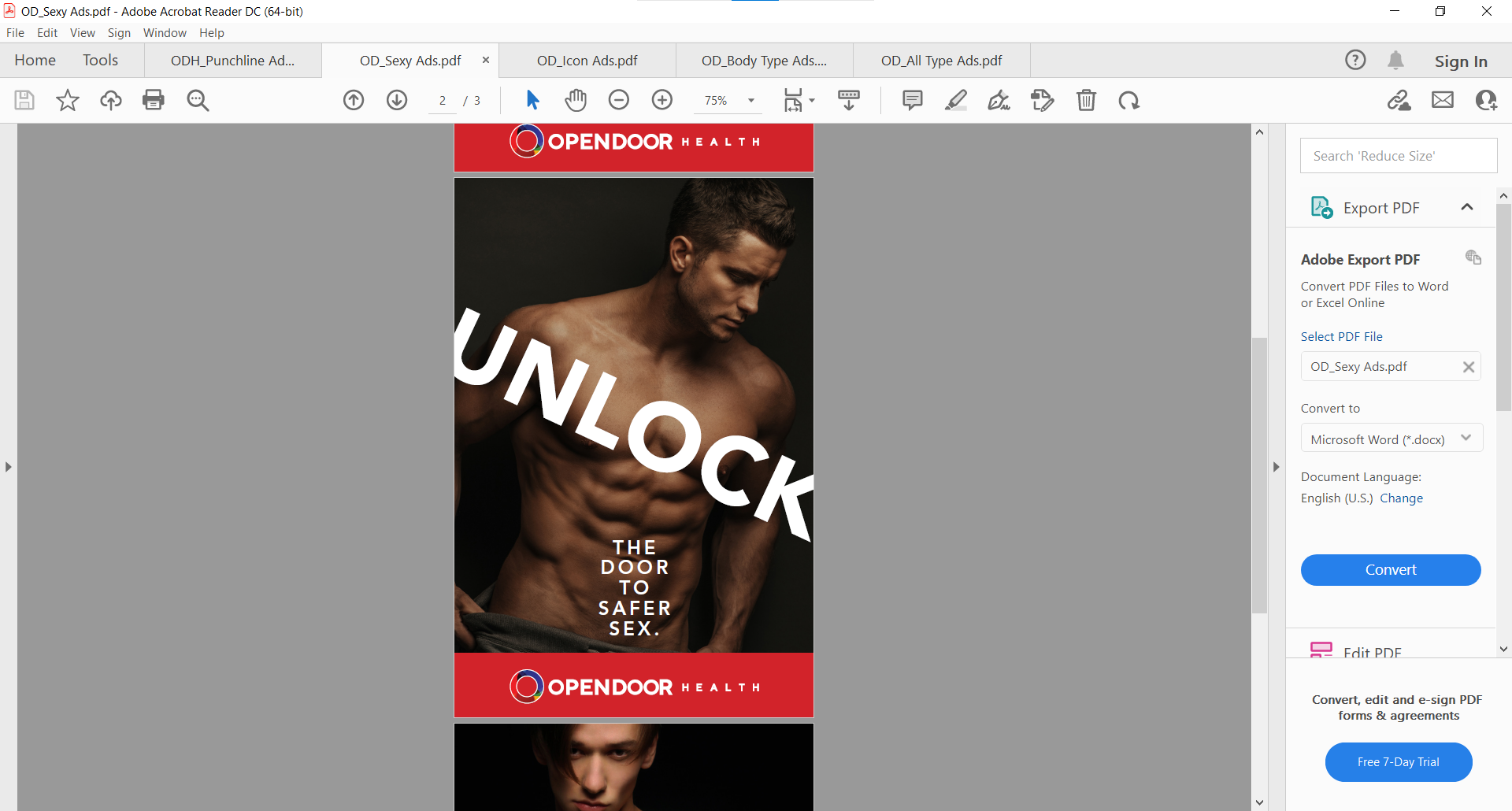


Figure S3. Specific advertisements within “Punchline” advertisement type


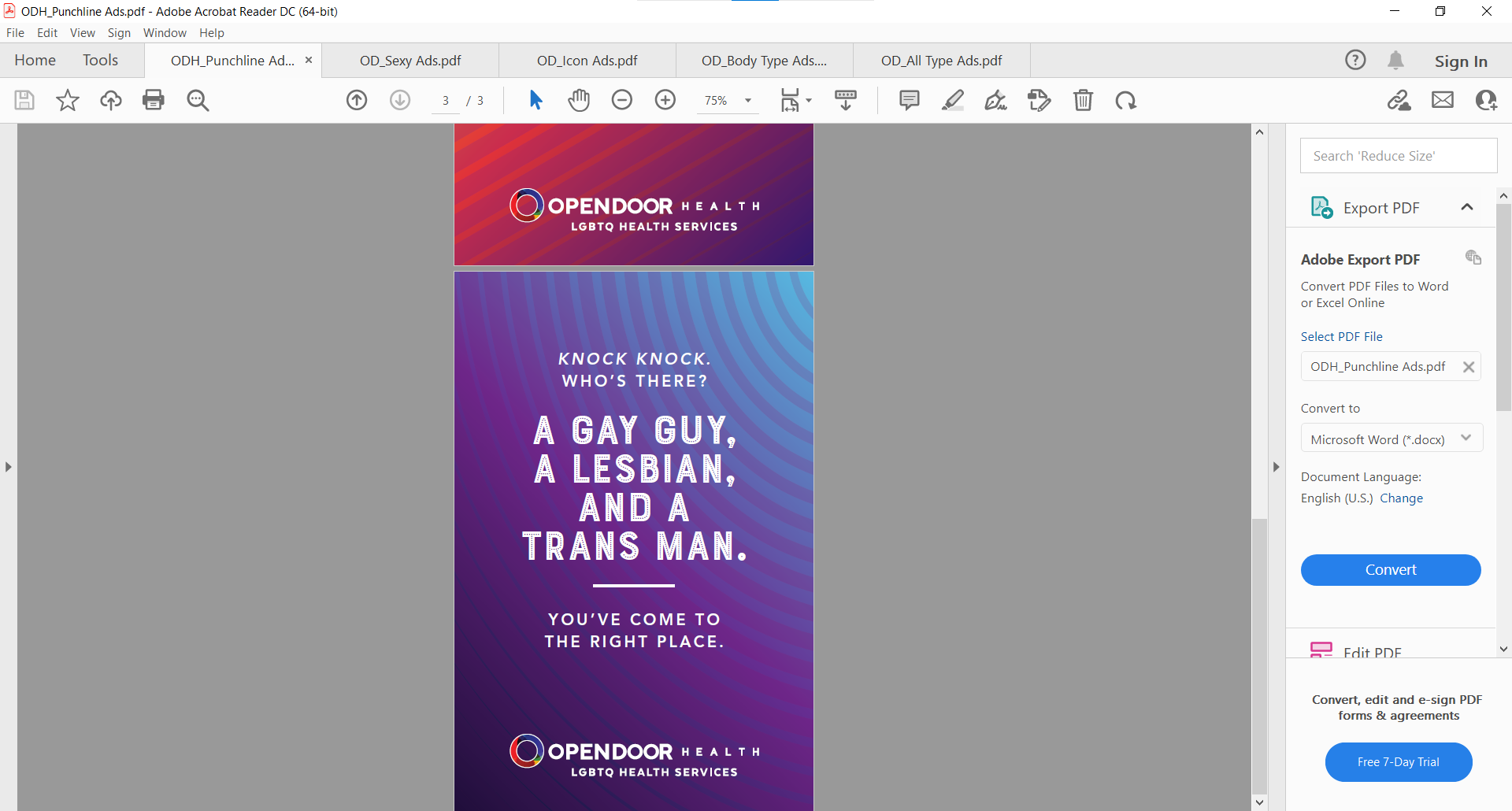

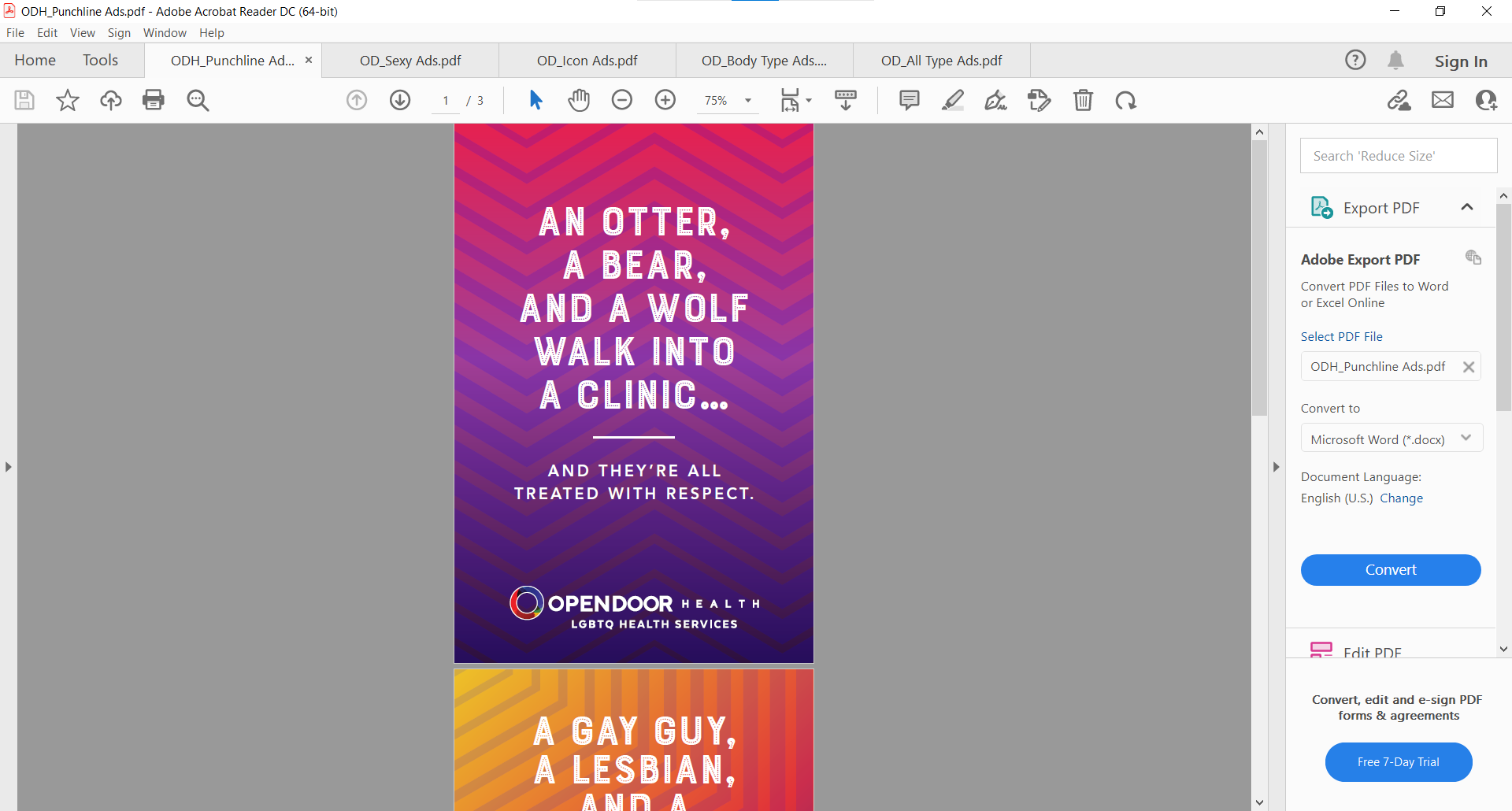

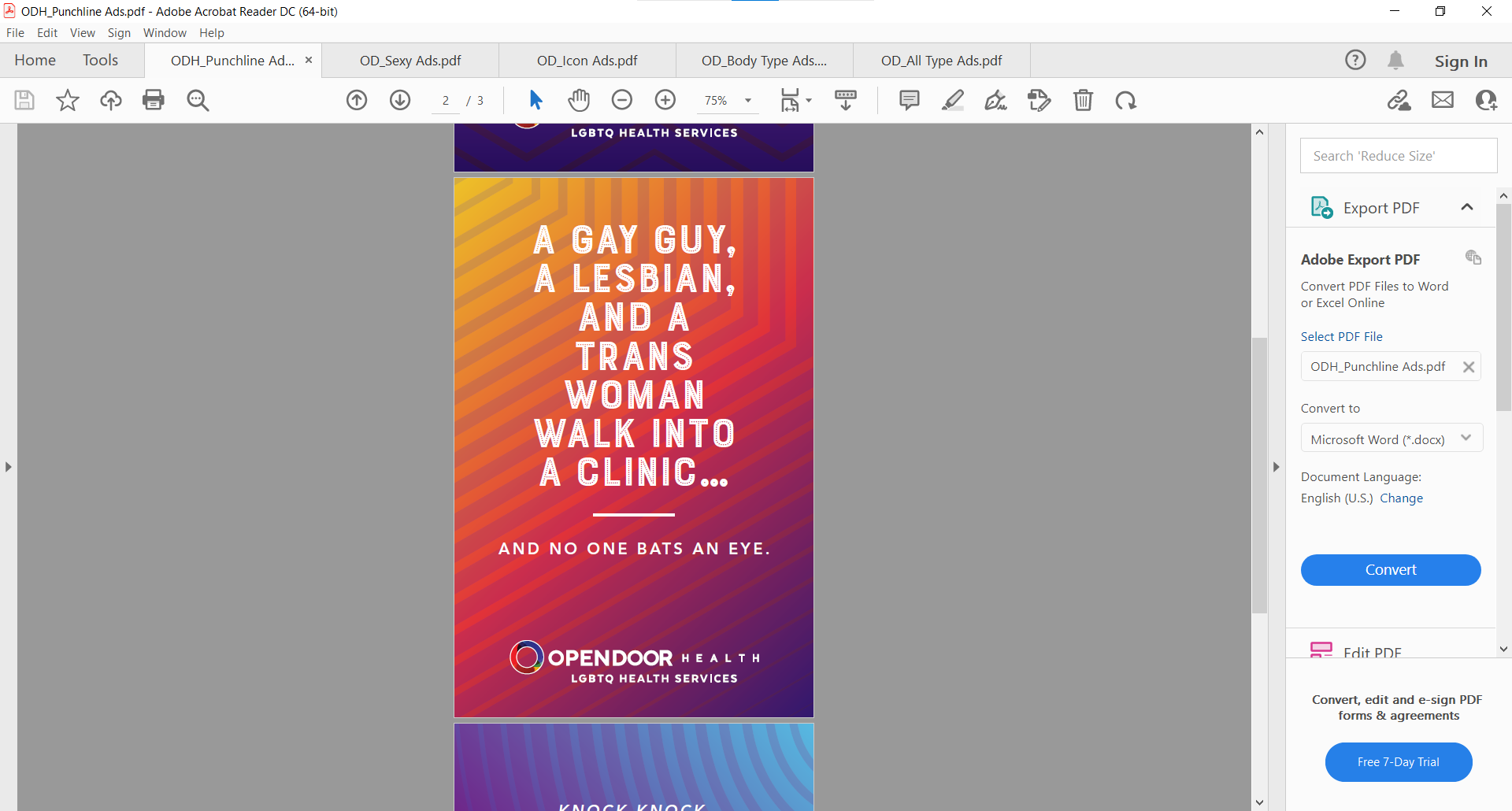


Figure S4. Example Google search campaign text-based advertisements


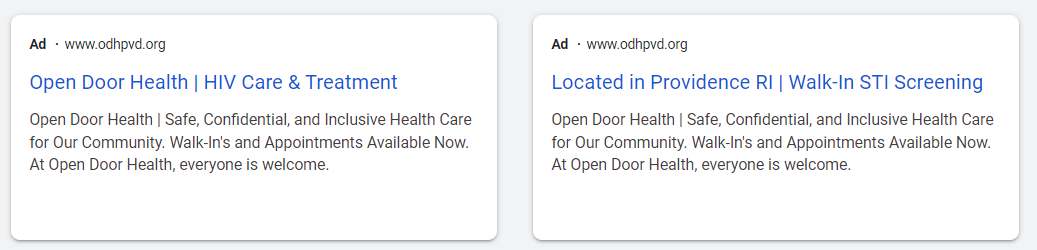

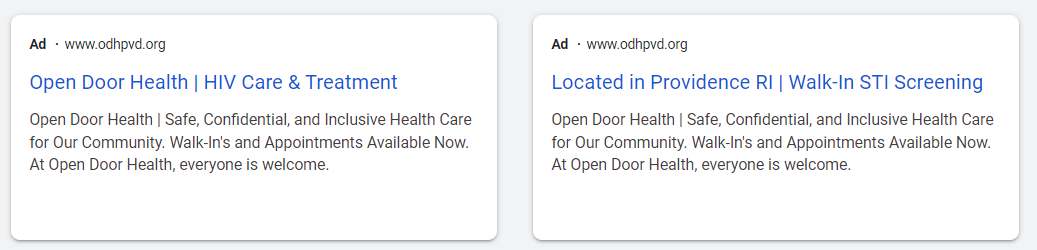


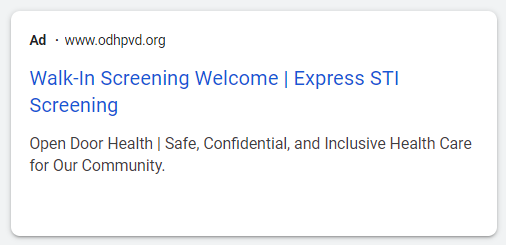

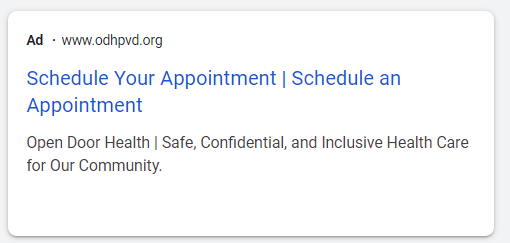


Figure S5. “Right Place” landing page


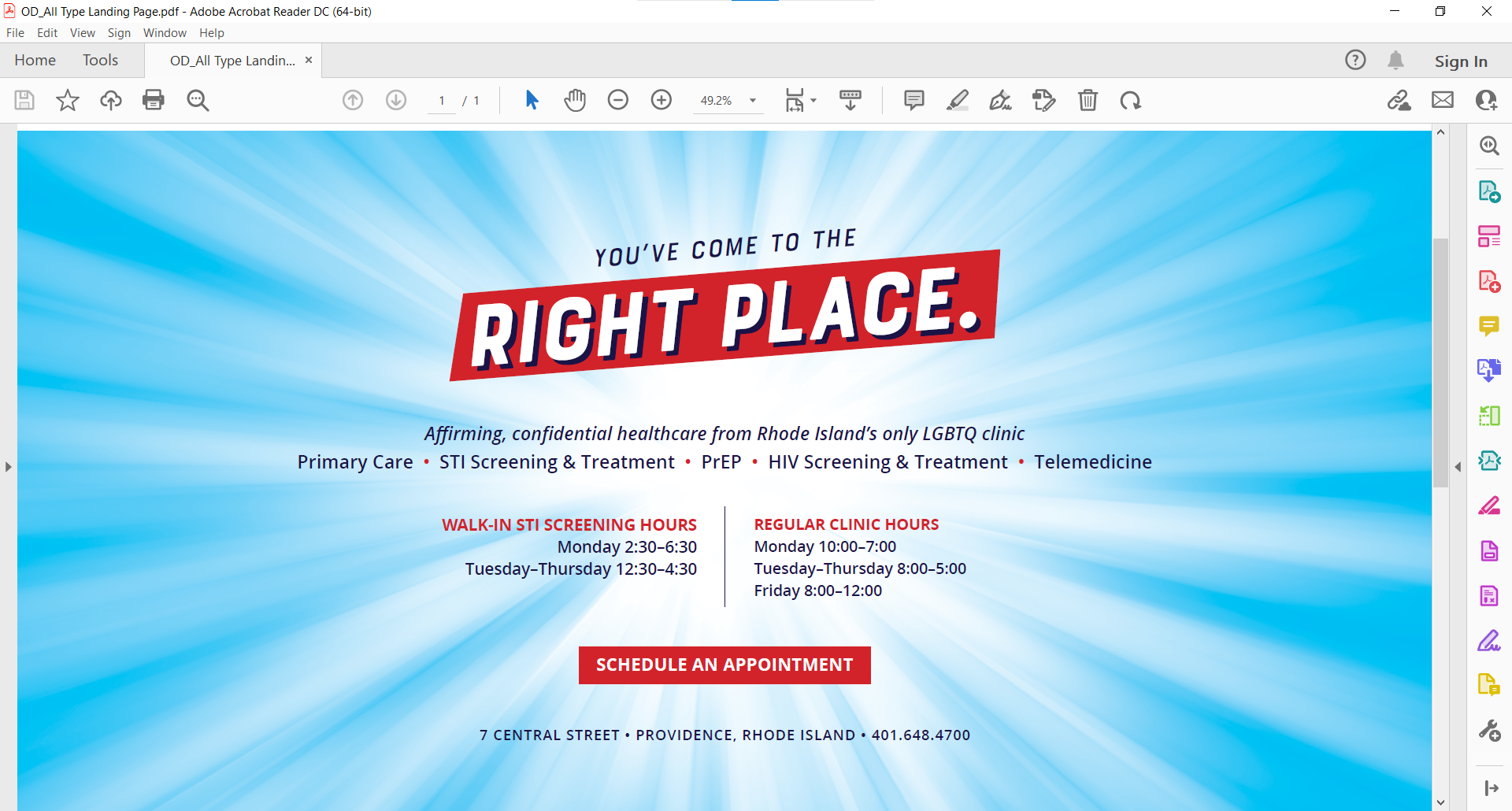


Figure S6. “Got You Covered” landing page


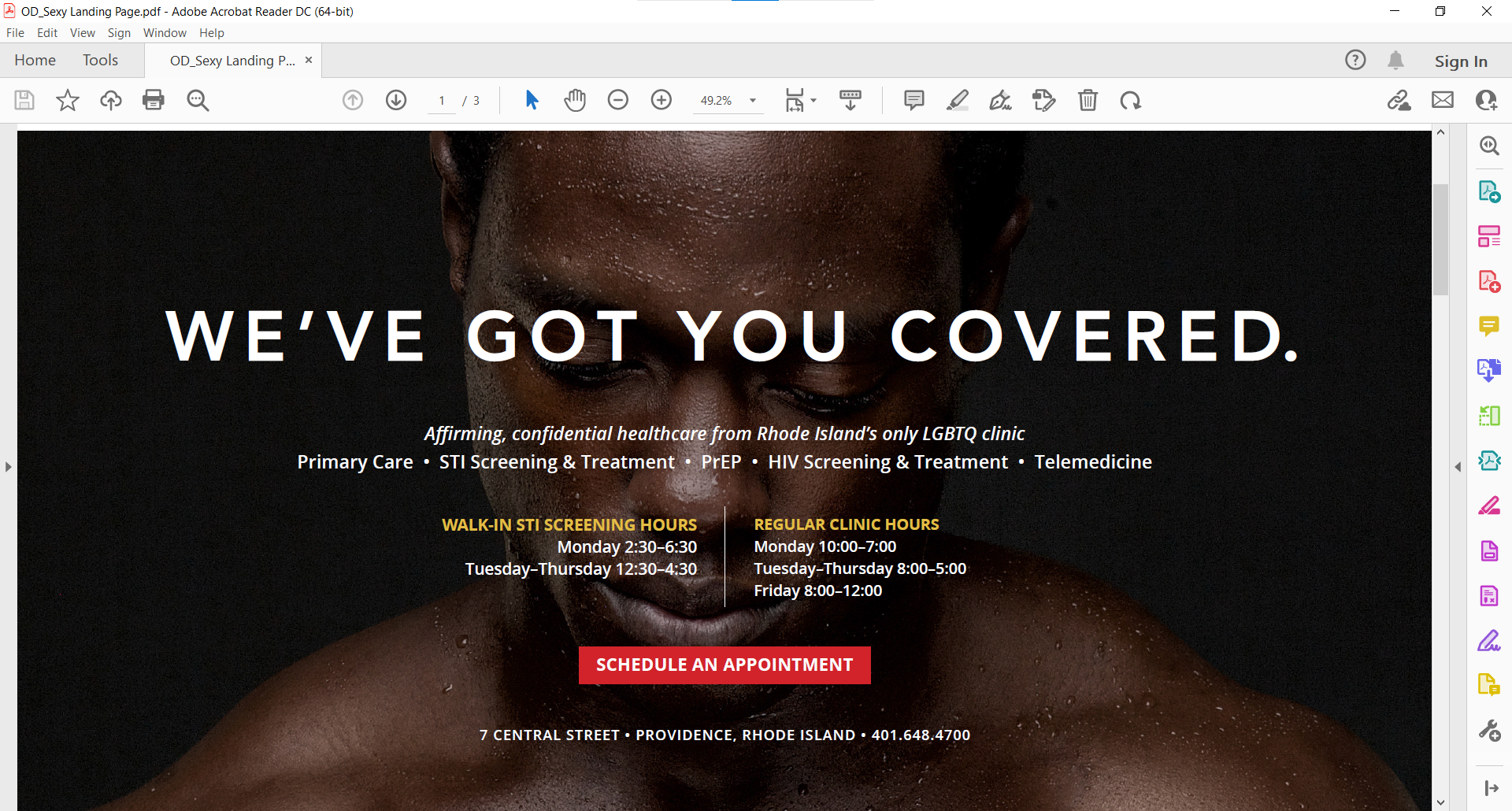


Figure S7. “Punchline” landing page


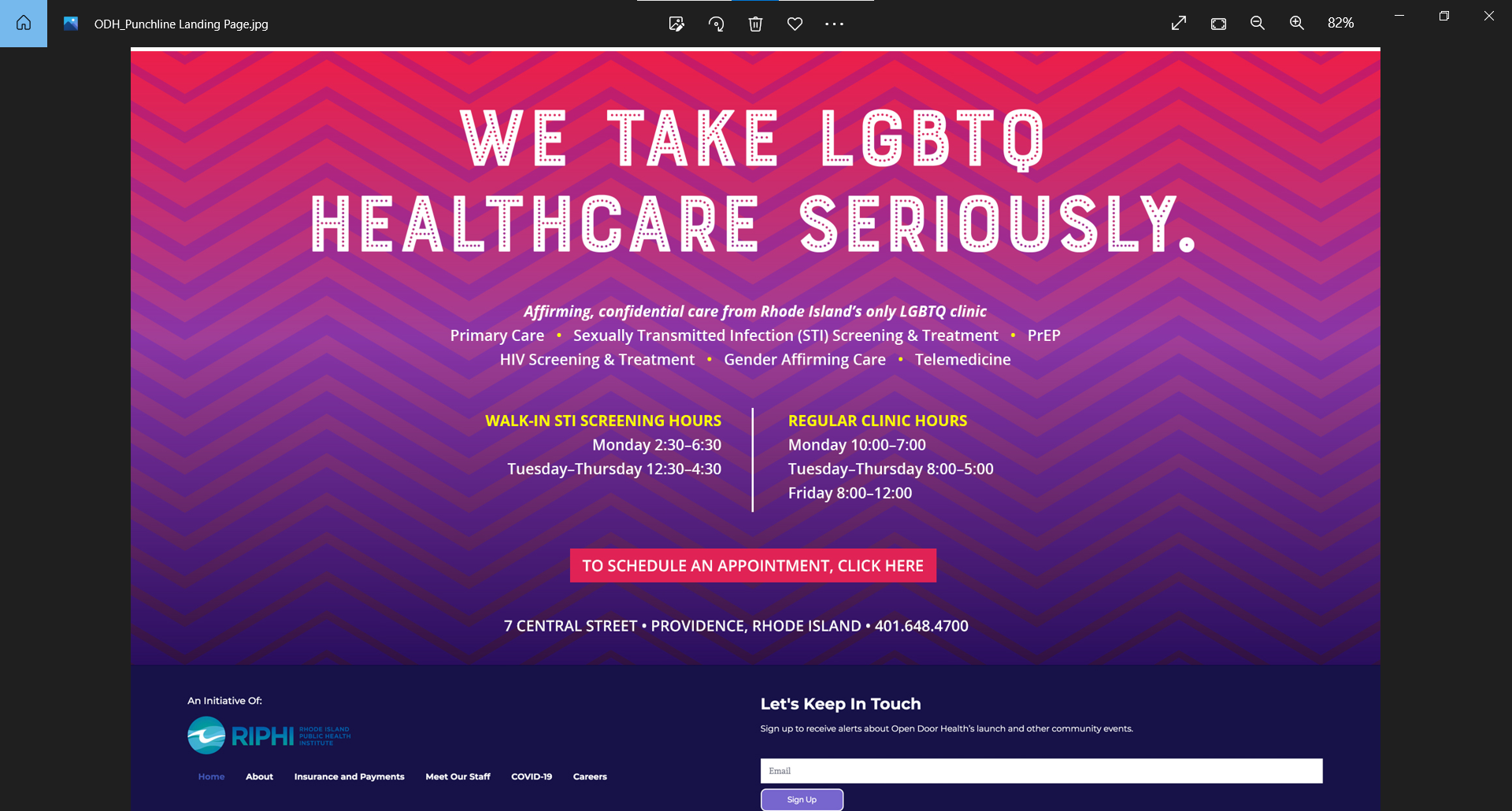


Table S1. Open Door Health “Right Place” campaign results, by specific advertisement and social media platform, April 1, 2021 – November 30, 2021

|  | Google – search* | Google – display | Grindr^†^ | Facebook |
| --- | --- | --- | --- | --- |
| “Right Place” - in and out | | | | |
| Reach | Not applicable | Not measured | Ran but not applicable | 59,970 |
| Impressions |  | 151,404 |  | 197,494 |
| Clicks |  | 577 |  | 528 |
| Conversions |  | 0 |  | 1 |
| Spend |  | $694.36 |  | $1,384.19 |
| Click-through rate |  | 0.38% |  | 0.27% |
| Conversion rate |  | 0.00% |  | 0.19% |
| Spend per 1,000 impressions |  | $4.59 |  | $7.01 |
| Spend per click |  | $1.20 |  | $2.62 |
| Spend per conversion |  | Undefined |  | $1,384.19 |
|  |  |  |  |  |
| “Right Place” - want it hard | | | | |
| Reach | Not applicable | Not measured | Ran but not applicable | 169,257 |
| Impressions |  | 59,997 |  | 821,712 |
| Clicks |  | 472 |  | 7,926 |
| Conversions |  | 0 |  | 1 |
| Spend |  | $489.52 |  | $5,538.83 |
| Click-through rate |  | 0.79% |  | 0.96% |
| Conversion rate |  | 0.00% |  | 0.01% |
| Spend per 1,000 impressions |  | $8.16 |  | $6.74 |
| Spend per click |  | $1.04 |  | $0.70 |
| Spend per conversion |  | Undefined |  | $5,538.83 |

* The Google search campaign included text-only advertisements that were not specific to an advertisement type. Although people who clicked on the text-only advertisement were taken to a landing page designed for one of the advertisement types overall, the landing page was not specific to any given ad within that advertisement type.

† Grindr “Right Place” campaign combined both specific advertisements into a single advertisement.

Table S2. Open Door Health “Got You Covered” campaign results, by specific advertisement and social media platform, April 1, 2021 – November 30, 2021

|  | Google – search* | Google – display | Grindr^†^ | Facebook |
| --- | --- | --- | --- | --- |
| “Got You Covered” - looking | | | | |
| Reach | Not applicable | Not measured | Ran but not applicable | Did not run |
| Impressions |  | 70,318 |  |  |
| Clicks |  | 799 |  |  |
| Conversions |  | 0 |  |  |
| Spend |  | $1,131.95 |  |  |
| Click-through rate |  | 1.14% |  |  |
| Conversion rate |  | 0.00% |  |  |
| Spend per 1,000 impressions |  | $16.10 |  |  |
| Spend per click |  | $1.42 |  |  |
| Spend per conversion |  | Undefined |  |  |
|  |  |  |  |  |
| “Got You Covered” - unlock | | | | |
| Reach | Not applicable | Not measured | Ran but not applicable | Did not run |
| Impressions |  | 161,075 |  |  |
| Clicks |  | 549 |  |  |
| Conversions |  | 1 |  |  |
| Spend |  | $641.08 |  |  |
| Click-through rate |  | 0.34% |  |  |
| Conversion rate |  | 0.18% |  |  |
| Spend per 1,000 impressions |  | $3.98 |  |  |
| Spend per click |  | $1.17 |  |  |
| Spend per conversion |  | $641.08 |  |  |

* The Google search campaign included text-only advertisements that were not specific to an advertisement type. Although people who clicked on the text-only advertisement were taken to a landing page designed for one of the advertisement types overall, the landing page was not specific to any given ad within that advertisement type.

† Grindr “Got You Covered” campaign combined both specific advertisements into a single advertisement.

Table S3. Open Door Health “Punchline” campaign results, by specific advertisement and social media platform, April 1, 2021 – November 30, 2021

|  | Google – search* | Google – display | Grindr | Facebook |
| --- | --- | --- | --- | --- |
| “Punchline” - no one bats an eye | | | | |
| Reach | Not applicable | Not measured | Did not run | 55,865 |
| Impressions |  | 139,947 |  | 145,342 |
| Clicks |  | 321 |  | 2,725 |
| Conversions |  | 0 |  | 0 |
| Spend |  | $292.86 |  | $1,725.13 |
| Click-through rate |  | 0.23% |  | 1.87% |
| Conversion rate |  | 0.00% |  | 0.00% |
| Spend per 1,000 impressions |  | $2.09 |  | $11.87 |
| Spend per click |  | $0.91 |  | $0.63 |
| Spend per conversion |  | Undefined |  | Undefined |
|  |  |  |  |  |
| “Punchline”- otter |  |  |  |  |
| Reach | Not applicable | Not measured | Did not run | 6,940 |
| Impressions |  | 110966 |  | 53,523 |
| Clicks |  | 172 |  | 175 |
| Conversions |  | 0 |  | 0 |
| Spend |  | $128.72 |  | $446.40 |
| Click-through rate |  | 0.16% |  | 0.33% |
| Conversion rate |  | 0.00% |  | 0.00% |
| Spend per 1,000 impressions |  | $1.16 |  | $8.34 |
| Spend per click |  | $0.75 |  | $2.55 |
| Spend per conversion |  | Undefined |  | Undefined |
|  |  |  |  |  |
| “Punchline” - knock knock | | | | |
| Reach | Not applicable | Not measured | Did not run | 9,968 |
| Impressions |  | 12,356 |  | 36,325 |
| Clicks |  | 40 |  | 198 |
| Conversions |  | 0 |  | 1 |
| Spend |  | $57.54 |  | $266.72 |
| Click-through rate |  | 0.32% |  | 0.55% |
| Conversion rate |  | 0.00% |  | 0.51% |
| Spend per 1,000 impressions |  | $4.66 |  | $7.34 |
| Spend per click |  | $1.44 |  | $1.35 |
| Spend per conversion |  | Undefined |  | $266.72 |

* The Google search campaign included text-only advertisements that were not specific to an advertisement type. Although people who clicked on the text-only advertisement were taken to a landing page designed for one of the advertisement types overall, the landing page was not specific to any given ad within that advertisement type.
